# Intraovarian Transplantation of Female Germline Stem Cells Rescue Ovarian Function in Chemotherapy-Injured Ovaries

**DOI:** 10.1371/journal.pone.0139824

**Published:** 2015-10-02

**Authors:** Jiaqiang Xiong, Zhiyong Lu, Meng Wu, Jinjin Zhang, Jing Cheng, Aiyue Luo, Wei Shen, Li Fang, Su Zhou, Shixuan Wang

**Affiliations:** 1 Department of Obstetrics and Gynecology, Tongji Hospital, Tongji Medical College, Huazhong University of Science and Technology, Wuhan, Hubei, China; 2 Hubei Key Laboratory of Embryonic Stem Cell Research, Tai-He Hospital, Hubei University of Medicine, Shiyan, Hubei, China; University of Connecticut, UNITED STATES

## Abstract

Early menopause and infertility often occur in female cancer patients after chemotherapy (CTx). For these patients, oocyte/embryo cryopreservation or ovarian tissue cryopreservation is the current modality for fertility preservation. However, the above methods are limited in the long-term protection of ovarian function, especially for fertility preservation (very few females with cancer have achieved pregnancy with cryopreserved ovarian tissue or eggs until now). In addition, the above methods are subject to their scope (females with no husband or prepubertal females with no mature oocytes). Thus, many females who suffer from cancers would not adopt the above methods pre- and post-CTx due to their uncertainty, safety and cost-effectiveness. Therefore, millions of women have achieved long-term survival after thorough CTx treatment and have desired to rescue their ovarian function and fertility with economic, durable and reliable methods. Recently, some studies showed that mice with infertility caused by CTx can produce normal offspring through intraovarian injection of exogenous female germline stem cells (FGSCs). Though exogenous FGSC can be derived from mice without immune rejection in the same strain, it is difficult to obtain human female germline stem cells (hFGSCs), and immune rejection could occur between different individuals. In this study, infertility in mice was caused by CTx, and the ability of FGSCs to restore ovarian function or even produce offspring was assessed. We had successfully isolated and purified the FGSCs from adult female mice two weeks after CTx. After infection with GFP-carrying virus, the FGSCs were transplanted into ovaries of mice with infertility caused by CTx. Finally, ovarian function was restored and the recipients produced offspring long-term. These findings showed that mice with CTx possessed FGSCs, restoring ovarian function and avoiding immune rejection from exogenous germline stem cells.

## Introduction

According to the 2012 GLOBOCAN statistics, approximately 8.2 million cancer patients died and 14.1 million new cases of cancer were diagnosed in 2012 worldwide. Nevertheless, the number of cancer survivors continues to increase due to the growth of the population and improvements in medical standards [1]. Furthermore, according to the American Cancer Society and the National Cancer Institute, there were approximately 14.5 million cancer patients on January 1, 2014 in America, and this population is expected to grow to almost 19 million in ten years [2]. The long-term side effects of exposure to chemotherapy treatments in women often lead to damage to ovarian function and can even induce amenorrhea as well as infertility [3–5]. During the past several decades, very little progress has been made to protect ovarian function and fertility during chemotherapy. A huge breakthrough for ovarian function protection and fertility preservation will not occur unless a new promising method of transplantation of FGSCs appears. Fortunately, it has been reported recently that FGSCs can play a significant role in this breakthrough. The traditional paradigm is that the limited oocyte-containing follicles cannot be renewed after birth and will be gradually exhausted until amenorrhea [6]. However, in 2004, Tilly et al.[7] reported that the number of follicles within the ovary is not fixed, as the decreasing rate of the existing number of follicles is inconsistent with the increased rate of the number of atretic follicles, which indicates that FGSCs may exist. In 2008, Zou et al.[8] successfully isolated and purified FGSCs from neonatal and adult mouse ovaries. After long-term in vitro culture, the FGSCs were infected with a GFP-carrying virus and were transplanted into ovaries, when the female mice receiving the transplant were mated with male mice, they produced some offspring that had the GFP transgene. Several years later, Tilly et al.[9] successfully isolated the hFGSCs from women of reproductive age. However, some researchers found no evidence of in vivo oocyte renewal from the putative FGSCs, and they therefore questioned the nature of the reported FGSCs [10,11]. Although controversy exists, stem-cell transplantation can be used as a novel method for the treatment of ovarian failure [12]. Because there are so many women, especially young females, who undergo chemotherapy and suffer from ovarian damage, we sought to determine whether FGSCs exist and whether stem-cell transplantation could be considered as a therapeutic option to restore ovarian function and infertility caused by CTx using a mouse model of CTx-induced ovarian failure.

## Materials and Methods

### Animal Model

All 59 mice used in these studies were C57BL/6 and purchased from the Center of Medical Experimental Animals of Hubei Province (Wuhan, China). Animals from 3 to 7 weeks of age were used. All mice were housed in specific pathogen-free (SPF) conditions with free access to the standard rodent diet and water. Ovarian failure was induced in the seven-week-old mice by a single intraperitoneal injection of busulfan (30 mg/kg) plus cyclophosphamide (120 mg/kg) as described previously [8]. Controls were injected with dimethyl sulfoxide (DMSO). The experiments were approved by the Animal Care Committee of the Tongji Medical College at the Huazhong University of Science and Technology in China.

### Isolation and culture of FGSCs

Eight ovaries were collected from four 6-week-old adult mice two weeks after CTx (busulfan 30 mg/kg, cyclophosphamide 120 mg/kg), and the FGSCs were isolated using methods described previously [8,9,13]. Briefly, the ovaries from female mice were dissected, and a sterile ophthalmic scissor was used to cut them into a slurry in Hank’s balanced salt solution without calcium or magnesium (HBSS) containing collagenase/Dnase I (Type IV; Sigma); the slurry was then incubated at 37°C with intermittent shaking for 30 min. The ovarian tissue was resuspended in HBSS and centrifuged two times in order to remove collagenase, followed by 0.05% trypsin treatment incubated at 37°C for 10 min. Finally, the trypsin was neutralized by adding 10% fetal bovine serum (FBS). The suspension was centrifuged, and the supernatant was carefully removed from the pellet. The cells were resuspended in FGSCs culture medium and plated on one well of a six-well plate. About three or four days later, when the cell density reached confluence, the cells were trypsinized and purified by magnetic activated cell sorting (MACS) using Fragilis antibody (Abcam) and goat anti-rabbit IgG microbeads (Miltenyi Biotec) according to the manufacturer’s instructions. The sorted cells enriched with FGSCs were plated on one well of a 24-well plate with the defined medium, which consisted of minimum essential medium α (MEM-α), 10% FBS, 1 mM non-essential amino acids, 1 mM sodium pyruvate, 0.1 mM β-mercaptoethanol (Sigma), 10 ng/mlLIF (Millipore), 10 ng/ml EGF (mouse epidermal growth factor; Sigma), 20 ng/ml human GDNF (glial cell line-derived neurotrophic factor; R&D systems), 1 ng/ml human bFGF (basic fibroblast growth factor; BD Biosciences), 1×concentrated N^2^-supplement and 1×concentrated penicillin-streptomycin. Feeder cells were needed to support the growth and proliferation in the early stage FGSC culture. When the FGSCs were stably established, the subsequent subculture of FGSCs did not need feeder cells, as the cell density of FGSCs was appropriate during the culture on the cell plates. The medium was changed every 2–3 days, and cells were subcultured every 3–8 days at a 1:1–3 dilution. All cultures were maintained at 37°C in a 5% CO_2_ atmosphere. In addition, the FGSCs were also isolated from 6 prepubertal female mice (3-week old) without chemotherapy treatment according to the above method.

### Immunofluorescence

Immunocytochemical staining was performed to analyze cultured cells and to determine their stemness. The staining was performed as follows: FGSCs were fixed with 4% paraformaldehyde for 15 min at room temperature, washed in PBS three times and then incubated in blocking solution (10% normal goat serum in PBS) at 37°C for 1 h followed by incubation at 37°C for 1 h with primary antibodies: rabbit polyclonal anti-Mvh (1:200 dilution, Abcam) and rabbit polyclonal anti-Fragilis (1:500 dilution, Abcam). After three washes in PBS, the FGSCs were incubated with FITC-conjugated secondary antibody (goat anti-rabbit IgG, 1:200 dilution) in the dark, then stained by Hoechst for 15 min. The FGSCs were washed in PBS three times and then finally observed under fluorescence microscopy.

### Reverse transcription-polymerase chain reaction

Total RNA was extracted with TRIzol reagent (Qiagen, Valencia, CA, USA) according to the manufacturer’s instructions. The RNA concentration was detected using the ND–1000 spectrophotometer (NanoDrop Technologies, USA), the residual DNA was treated with DnaseI, and approximately 2 μg of RNA was used to synthesize cDNA by reverse transcriptase following the manufacturer’s recommended protocol (Transcriptor cDNA first strand synthesis kit, Roche). Amplification was performed for each gene with specific primer sets as described by Wu et al.[8], and the glyceraldehyde-3-phosphate dehydrogenase gene (GAPDH) was used as a loading control.

### Karyotype analysis of FGSCs

After 3 days of FGSC passage, the cells that were cultured for more than 15 generations were treated with FGSC culture medium supplemented with colchicine (80 ng/ml) for 3 h. They were then hypotonically treated with 40 mM KCl for 30 min and fixed in methanol-acetic acid (3:1) for 1 h. The treated FGSCs were dropped on the slides from a certain height, air-dried, stained with Giemsa buffer and observed under the oil microscope.

### Lentiviral transfection of FGSC lines and identification by flow cytometry

Before the transfection, FGSCs were plated on one well of a six-well plate. When the cells reached the logarithmic phase, 1mgMSCV-PUbi-GFP vector (Genechem Company, Shanghai, China) was transfected into the cells according to the manufacturer’s instructions. The cells were incubated in transfection medium for 12–18 h followed by three washes and then cultured in FGSC culture medium. Three days later, the expression of GFP was observed under fluorescence microscopy. After being cultured for several passages, some of the FGSCs were used to detect the transfection efficiency of GFP-carrying virus by flow cytometry.

### Self-inactivation of lentivectors in FGSCs

To confirm whether GFP-infected FGSC lines were unable to generate infectious lentiviral particles, stable FGSC lines transduced with MSCV-PUbi-GFP vector were seeded into one well of a six-well plate with a confluence of approximately 30% and were cultured for 3 days to nearly 75% without changing the culture medium. The supernatant was collected and filtered with a 0.45 μm nylon membrane. Then, the filtered supernatant was incubated with non-transduced FGSC lines, and the experiments were performed in triplicate. After being cultured for three days, no GFP expression was detected in FGSC lines incubated in supernatant, demonstrating that MSCV-PUbi-GFP lentiviral particle-transduced FGSC lines do not release infectious lentiviral particles into the medium.

### FGSCs transplantation into recipient mice

After transfection with GFP-carrying virus, the FGSCs were trypsinized, neutralized by 10% FBS, washed with PBS and resuspended in culture medium. Recipients were anaesthetized with 1% sodium pentobarbitone injection (intraperitoneal). A single-cell suspension of 3μl, containing approximately 2 × 10^4^ cells, was injected into ovaries using a 10-μl NanoFil syringe with a 33-gauge beveled needle (World Precision Instruments).

### Southern blotting

DNA probes were synthesized by PCR amplification from plasmid DNA carrying the GFP gene as a template using the following specific primers: 5’-ATGGTGAGCAAGGGCGAGG–3’ and 5’-CGTCCTCGATGTTGTGG–3’. Digoxigenin labeling was used to detect the GFP gene. Genomic DNA was extracted from the tails of the progenies with the Genomic DNA extraction kit according to the manufacturer’s instructions (Qiagen) and digested with PstI. The digested DNA was loaded into0.8% agarose gels, and plasmid DNA was used as a positive control. After running for several hours, the separated DNA fragments were transferred to 0.45 μm nylon membranes, and subsequent hybridization and stringency washes were performed. Finally, the detection was performed using anti-Digoxigenin Alkaline phosphatase antibody conjugate according to DIG Detection Kit (Roche) following the manufacturer’s manual.

### Enzyme immunoassay for 17β-estradiol

Six female mice without any treatment constituted Group1A; six female mice that were administered nonlethal doses of CTx (busulfan 30 mg/kg, cyclophosphamide 120 mg/kg) at 3weeks old and transplanted with FGSCs at 6-weeks old constituted Group1B, and another seven female mice administered the same dose of CTx at 3-weeks old and injected with the same volume of PBS at 6-weeks old constituted Group1C. They were all purchased at 3-weeks old and were housed until they were 8-weeks old before they were killed to determine the serum estrogen concentration. The concentration of 17β-estradiol in serum was determined using an Estradiol EIA kit (Cayman Chemical Company, Ann Arbor, USA) according to the manufacturer's instructions. Briefly, 50μl of the serum samples was incubated in microtiter wells coated with goat anti-mouse monoclonal antibody against estradiol. After incubation and washing, the concentration of 17β-estradiol was determined using aspectrophotometer (Bio-Tek, Winooski, USA).

### Statistical Analysis

The Mann-Whitney U test was performed to analyze the significant differences between the groups for their total number of offspring over the course of the 4-month mating trials, and one-way analysis of variance was used to assess the difference in the serum concentration of estrogen. All the statistical methods were performed with SPSS. Significance was accepted at *P*<0.05.

## Results

### Isolation and culture of FGSCs

**Fig 1:**
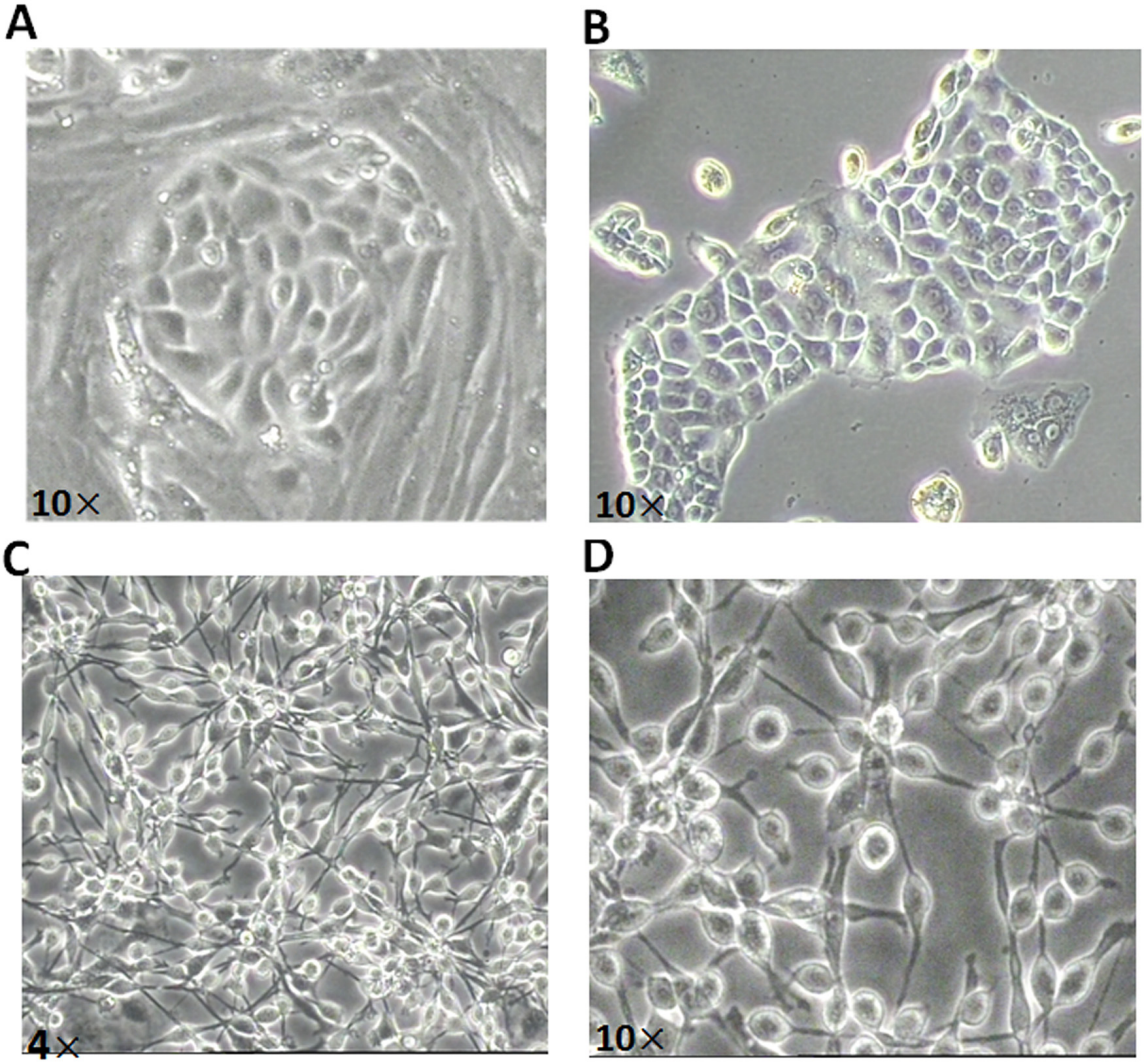
The morphology and characteristics of FGSCs. (A) The morphological characteristics of FGSCs before purification. (B) The characteristics of FGSCs showed large cell bodies with little cytoplasm and the FGSCs grew in clusters during early culture after purification. (C-D)The cell morphology of FGSCs changed to a grape-like shape after many passages of culture *in vitro*.

**Fig 2:**
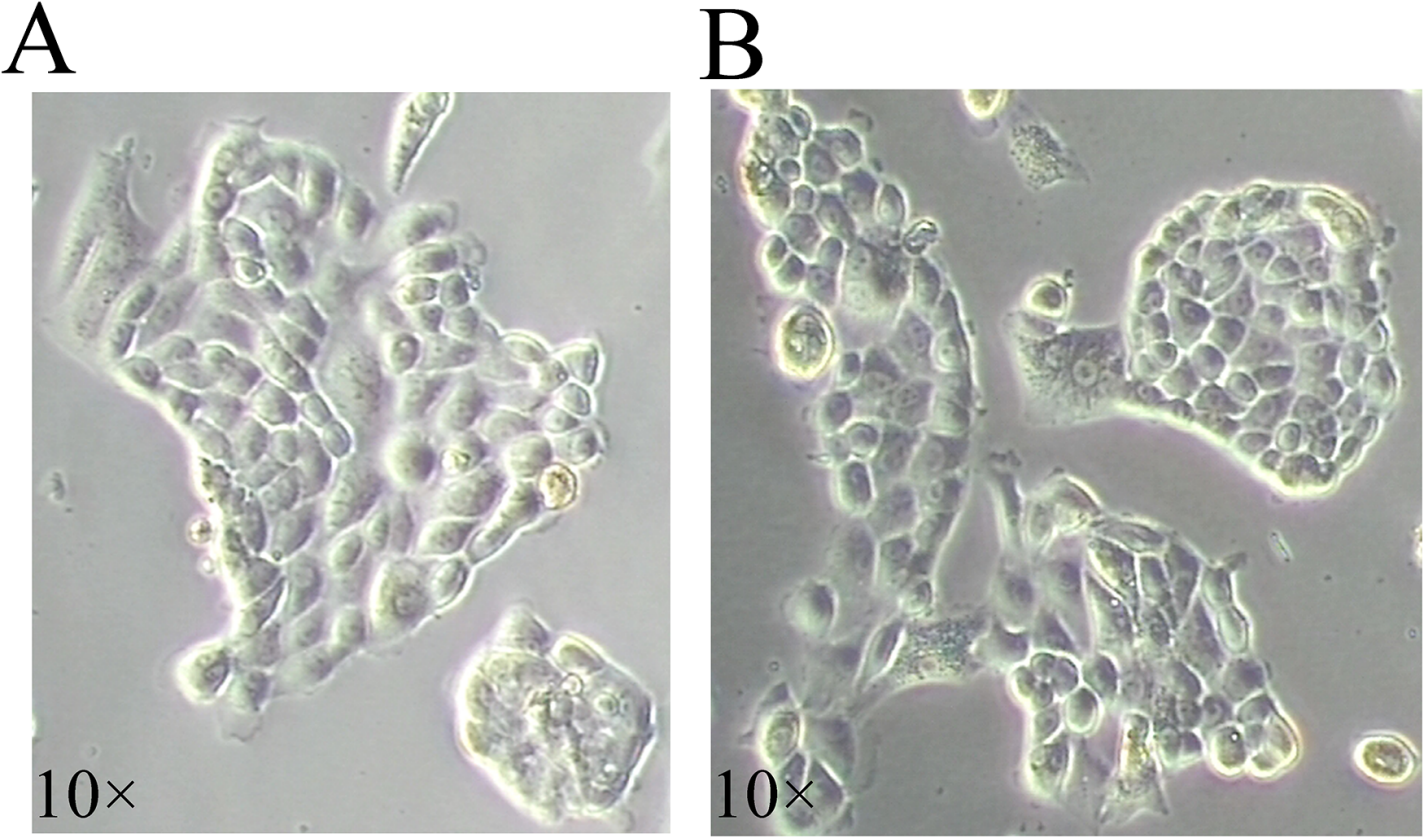
The morphology and characteristics of FGSCs isolated and purified from the prepubertal ovaries without chemotherapy. (A-B) The fresh isolated FGSCs grew in clusters duringearly culture after the purification.

Two weeks after the treatment with CTx mentioned above, four adult mice were anaesthetized to harvest ovaries. After a two-step enzymatic digestion of ovaries, a total of approximately 2×10^5^ cells was acquired, which contained granulosa cells, theca cells, trace oocytes and a small amount of FGSCs. Considering the small number of the separated cells and the inevitable damage during the process of isolation, we cultured the digested cells from 8 ovaries for 3–4 days, when the cell density reached confluence on the six-well plate and with the cell number increasing to 8–10×10^5^; then these cells were used for MACS and 2% (1.6–2×10^4^) positive cells were obtained with the antibody of Fragilis. After purification and several passages, the FGSC line was stably established. The purified FGSCs grew in clusters during the early stage of the subculture, and in grape-like shapes for subsequent passages, with a high nuclear-to-cytoplasm ratio during the culture (Fig 1A–1D), which was consistent with previous reports. In addition, we also isolated and purified the FGSCs from the prepubertal ovaries without chemotherapy using the same method mentioned above. The result showed that the purified FGSCs grew in clusters at the early stage (Fig 2A and 2B), which was consistent with the morphology of adult FGSCs during the same period after isolation from ovaries. After the FGSCs were stably established in vitro, these cells could be cultured without the feeder cells, as in the study by Tilly et al.[9,13].

### Identification of FGSCs for gene expression, immunofluorescence, AP staining, and karyotyping

**Fig 3:**
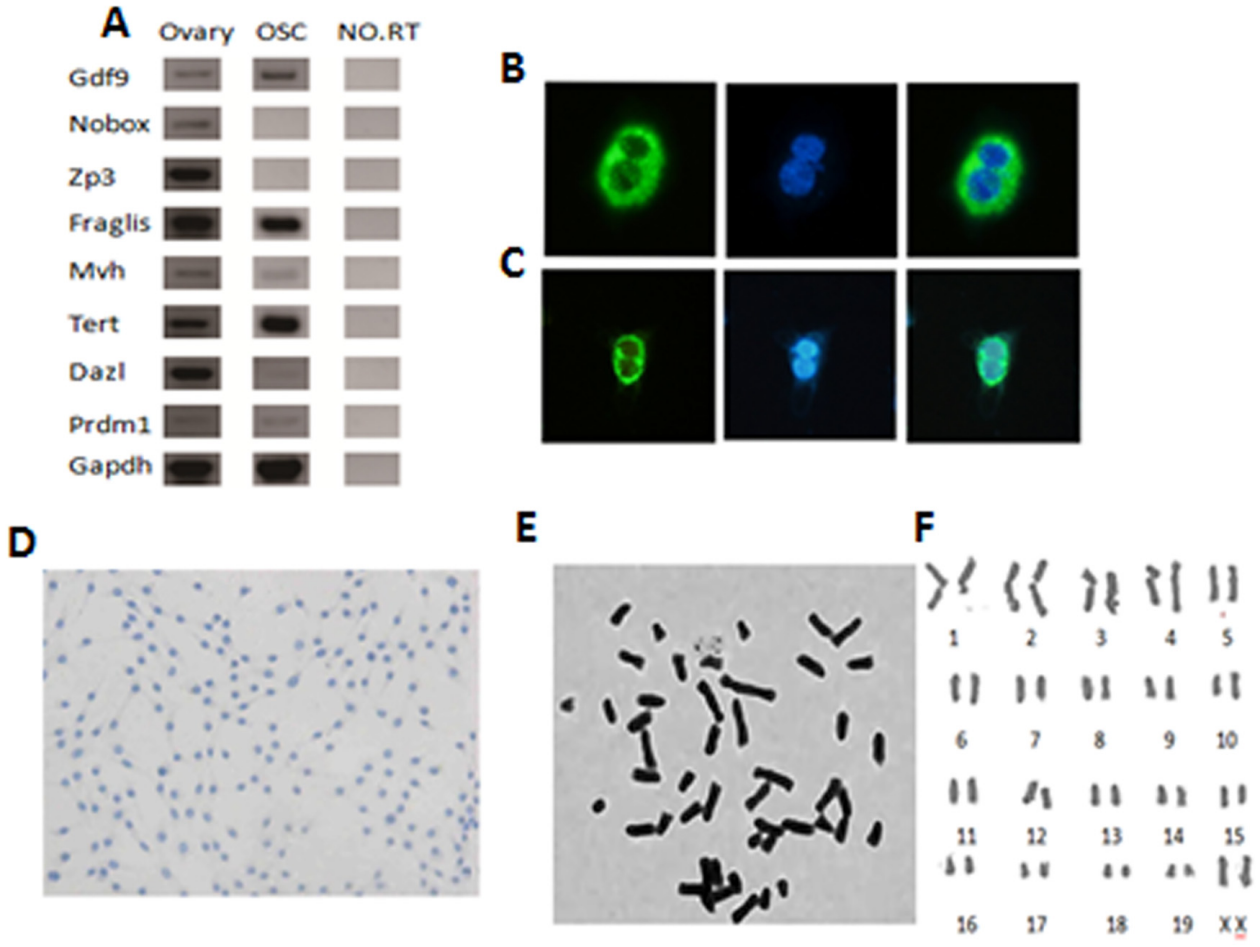
The identification of the established FGSCs. (A) Reverse transcriptional PCR analysis for the expression profile of FGSCs, controls and ovarian tissues. There were two set of genes: one for oocytes, including Gdf9, Nobox, and Zp3, and another for germ cells, including Fragilis, Mvh, Tert, Dazl and Prdm1. No RT, PCR of RNA samples without reverse transcription. (B-C) Immunofluorescence for Fragilis (above) and Mvh (below) in established FGSCs. Green, Fragilis and Mvh immunofluorescence; Blue, DAPI. (D) Alkaline phosphatase staining for established FGSCs. (E-F) Karyotype analysis of the FGSCs.

To confirm that the freshly isolated cells were FGSCs and not oocytes, we tested the expression of germ cell-and oocyte-specific genes: Fragilis, Mvh, Tert, Dazl, Prdm1, Gapdh, Nobox, Zp3 and Gdf9. The results showed that the specific genes of germline cells, including Fragilis, Mvh, Tert, Dazl and Prdm1, were all positive for FGSCs, while the specific genes for oocytes, including Nobox and Zp3, were negative (Fig 3A). These findings suggest that the established cells possessed the characteristics of germ cells. Subsequently, we detected the proteins encoded by the Fragilis and Mvh genes, which are classic primitive germline markers. The results showed that both Fragilis and Mvh were positive for the FGSCs (Fig 3B and 3C). Moreover, the FGSCs were weakly positive for alkaline phosphatase staining (Fig 3D), suggesting that the FGSCs were distinct from the embryonic stem cells (ESCs), which should be strongly positive for AP staining. Finally, we performed karyotyping of the FGSCs and the results displayed the normal karyotype in approximately 65% of cells (Fig 3E and 3F). In short, cultured FGSCs displayed the characteristics of female germline stem cells.

### Lentiviral transfection of FGSC lines

**Fig 4:**
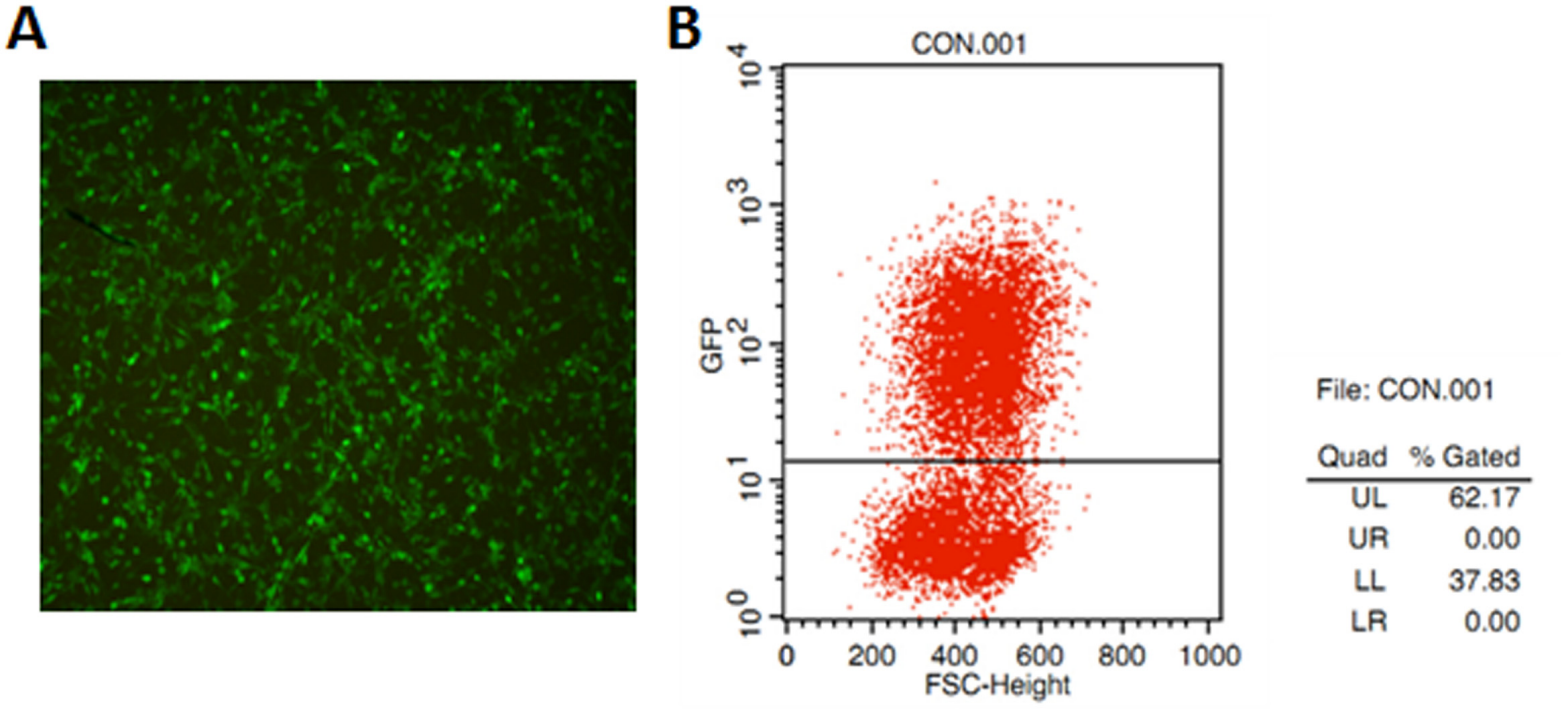
Lentiviral transfection of FGSC lines. (A) A majority of FGSCs were GFP positive after the lentiviral transfection under the fluorescence microscopy. (B) The detection of the transfection efficiency of FGSCs by flow cytometry.

Three days after transfection of the GFP-carrying virus, we found that a majority of the FGSCs were GFP-positive under fluorescence microscopy (Fig 4A). After several passages of the infected FGSCs, we detected the exact transfection efficiency of FGSCs by flow cytometry, which showed that 62.3% FGSCs were GFP-positive (Fig 4B). We passaged the infected FGSCs more than 18times to observe persistent GFP expression, and the results showed that the expression level of GFP remained comparably stable.

### FGSCs rescues ovarian function and long-term fertility

**Fig 5:**
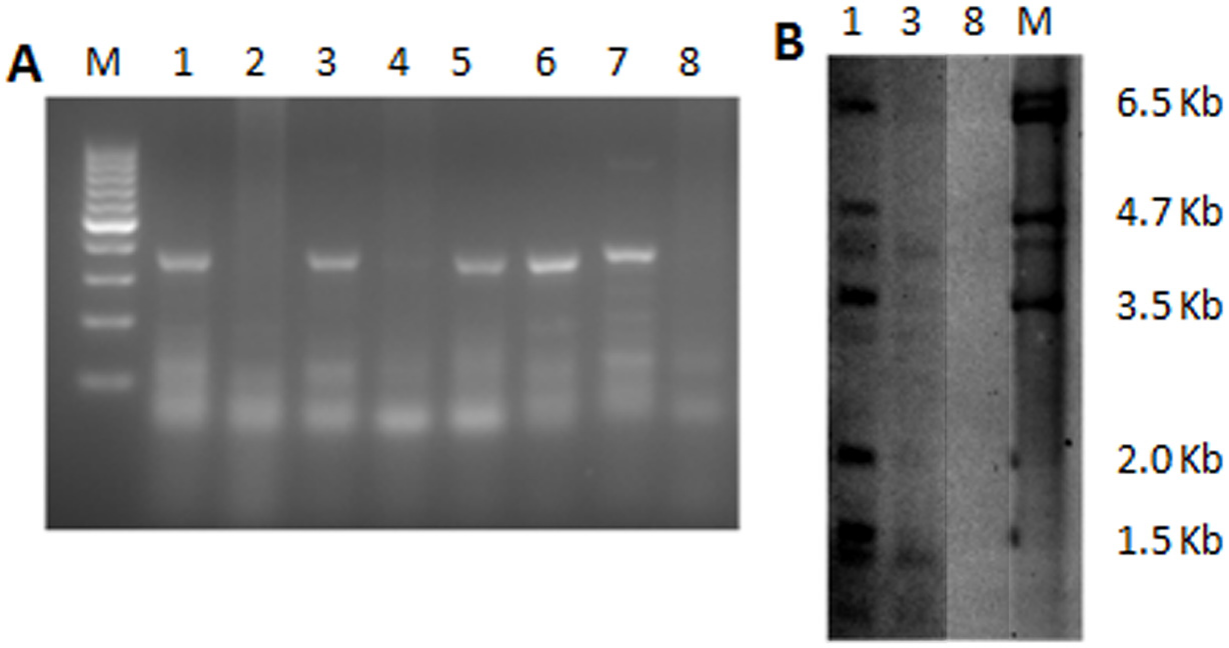
The detection of the GFP gene integrated into the genomes of offspring by PCR and Southern blot. (A) The GFP-positive samples were detected in the offspring by PCR. Samples 1–7 were offspring and sample 8 was wild-type. M: 100bp DNA ladder Marker; 1, 3, 5, 6 and 7 were positive for GFP, while 2, 4 and 8 were negative for GFP. (B) The further confirmation of GFP gene integrated into the genomes of offspring by Southern blot. M: the control plasmid; the offspring of samples 1 and 3 were positive for Southern blot and sample 8 from the wild-type mouse was negative.

During the four-month observation period, 6negative female controls without CTx (Group A) achieved three successful (live-birth) pregnancies (Table 1). However, females without FGSC transplantation (Group B), which were only administered nonlethal doses of CTx (cyclophosphamide 120mg/kg, busulfan 30 mg/kg), were found to be infertile when mated 5 weeks after CTx, with none achieving pregnancy (Table 1). Six females underwent FGSC injection 1week after nonlethal CTx (Group C) and mating 5 weeks after FGSCs transplantation. The results showed that 83.3% of the mice achieved three pregnancies (Table 1). The number of offspring produced in group B and C were significantly different (*P* < 0.05), while no significant difference existed between group A and group C (*P* = 0.257). To test whether the exogenous GFP gene was integrated into the genomes of FGSCs, the genome DNA from offspring, together with wild-type mice as a negative control, were extracted, and PCR screening was performed. The results showed that the majority of the offspring were GFP-positive (Fig 5A). To further confirm the integration of the GFP gene, two GFP-positive samples from offspring and one negative sample from wild-type mice were selected for Southern blot analysis (Fig 5B), which is consistent with the results of PCR. To assess the effect of time of FGSC transplantation on fertility outcome, 6 females were administered non-lethal doses of CTx (Group D), followed by the intraovarian injection of FGSCs 2 months after CTx, one week after the transplantation, the 6 females were then mated with males. The mating trial was consecutively observed for 4 months, and the results showed that none of the mice were pregnant (Table 1). Furthermore, to test whether the CTx dose was another important factor that could affect the ability of FGSCs to restore fertility, six females were administered median lethal doses (LD50) of CTx (cyclophosphamide 200 mg/kg, busulfan 20 mg/kg) without FGSC transplantation (Group E), while another six females were given the same CTx dose as Group E, but with FGSC transplantation1 week after CTx (Group F). Both Group E and F were mated with males 5 weeks after FGSC transplantation. In Group E, two mice died within 2.5 months and none of the mice had a pregnancy within 4 months. Compared to Group E, no mice died during the observation period, and no pregnancy was observed for 4 months in Group F (Table 1).

**Table 1:** The fertility results of each group over the course of the 4-month mating trial.

	Group A			Group B	Group C			Group D	Group E	Group F
Number	I	II	III	CO	I	II	III	CO	CO	CO
**Mice 1**	9	9	8	0	9	8	6	0	0	0
**Mice 2**	7	6	7	0	6	5	7	0	0	0
**Mice 3**	9	8	8	0	8	7	8	0	0	0
**Mice 4**	8	8	6	0	8	6	6	0	0	0
**Mice 5**	7	8	7	0	0	0	0	0	0	0
**Mice 6**	6	6	7	0	7	8	5	0	0	0

Note: Group A: negative female controls without CTx treatment; Group B: female mice administered nonlethal doses of CTx (busulfan 30 mg/kg, cyclophosphamide 120 mg/kg) without transplantation of FGSCs; Group C: female mice administered the same dose of CTx as Group B, with the transplantation of FGSCs one week after the CTx; Group D: female mice administered the same dose of CTx as Group B, with the transplantation of FGSCs two months after the CTx; Group E:female mice administered median lethal doses (LD50) of CTx (busulfan 20 mg/kg, cyclophosphamide 200 mg/kg), without the transplantation of FGSCs; Group F: female mice administered the same dose of CTx as Group E, with the transplantation of FGSCs one week after the CTx. I: the offspring number of the first generation; II: the offspring number of the second generation; III: the offspring number of the third generation; CO: the offspring number during continuous observation over the course of the 4-month mating trial.

### Evaluation of ovarian function upon serum hormone level

**Fig 6:**
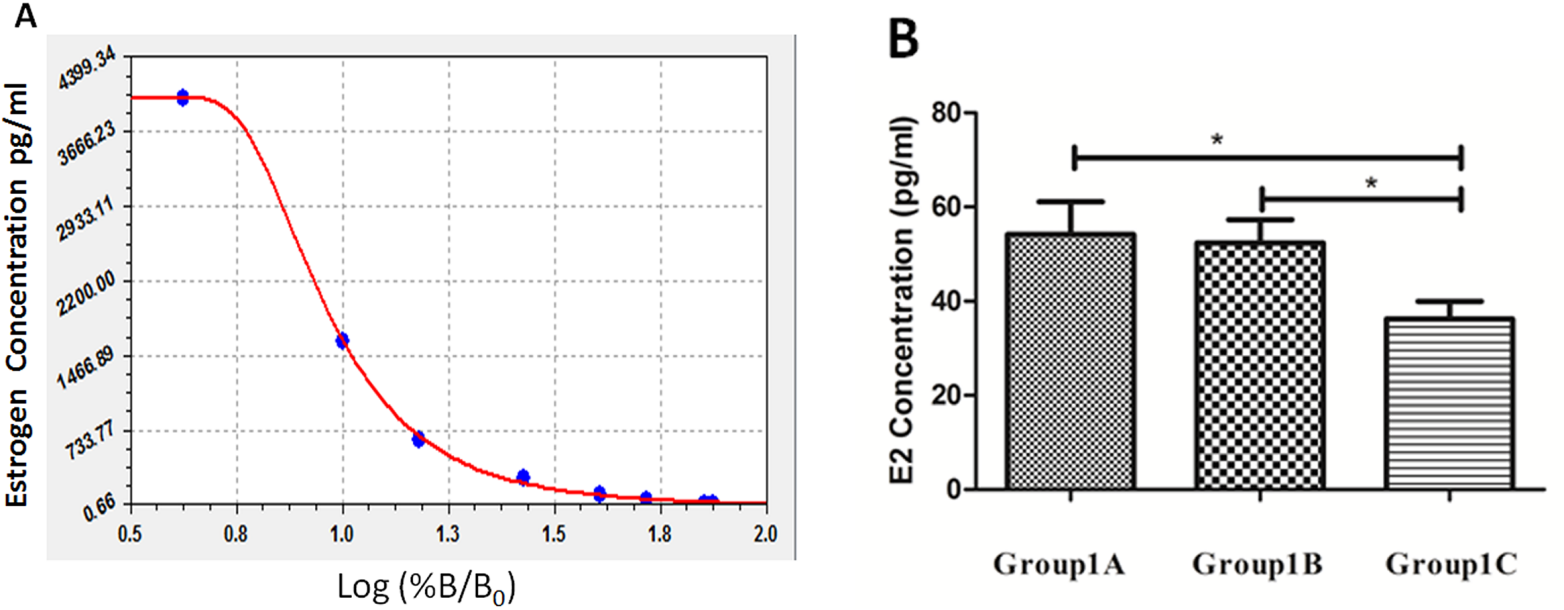
Serum estrogen concentration. (A) The standard curve obtained according to the Estradiol EIA kit. (B) There were significant differences between Groups 1A and 1C and between Groups 1B and 1C. The symbol * represents a significant difference.

According to the Estradiol EIA kit instruction, we obtained a standard curve as shown in Fig 6A. After the detection of serum estrogen concentrations of Group1A, 1B and 1C (Table 2), we performed statistical analysis and found that there was a significant difference between Group1A and Group1C (*P* = 0.027); there was also a significant difference between Group1B and Group1C (*P* = 0.043). However, no significant difference was found between Group1A and Group1B (*P* = 0.819). The statistical results are shown in Fig 6B.

**Table 2:** Serum estrogen levels of each group.

	Estrogen concentrations (pg/ml)						
Group 1A	43.29	36.83	66.92	82.28	45.74	49.84	
Group 1B	70.77	37.41	42.79	48.97	54.7	59.63	
Group 1C	45.13	30.7	40.63	33.93	49.14	34.99	19.02

Note: Group 1A: six normal female mice without any treatment; Group 1B: six female mice were administered with nonlethal doses of CTx (busulfan 30 mg/kg, cyclophosphamide 120 mg/kg) at 3-weeks old and then transplanted with FGSCs at 6-weeks old. Group 1C: seven female mice were administered the same dose of CTx as Group 1B at 3-weeks old, but were injected with the same volume of PBS at 6-weeks old. Mice in the above three groups were killed during diestrus for the detection of estrogen at 8-weeks old.

## Discussion

Improved therapies for cancer have resulted in a growing population of long-term survivors. Identifying the best way to improve the quality of life of survivors in their pre-reproductive or reproductive years is a serious problem [14]. In 2004, Tilly et al. first proposed that FGSCs also exist in female mammals, just like spermatogonial stem cells (SSC) exist in the testis, which was proven many years ago. However, this study challenged the traditional belief that the oocyte reserve endowed at birth is fixed and non-renewable, and several researchers have reported that no active FGSCs exist in adult mammalian ovaries in vivo [10,11,15,16]. Nonetheless, a number of studies have verified that FGSCs exist in adult mammalian ovaries and could be isolated and cultured in vitro for more than 15 months [8,13,17,18]. Recently, a stem cell-based treatment for the protection of ovarian function and oocyte formation has been proposed as a future clinical therapy to treat infertility in female animals [9,12,19,20]. However, whether the ovaries of women treated with CTx also contain FGSCs and whether the aforementioned FGSCs could be used to protect gonadal function and fertility remains unknown. To evaluate the possibility of autologous transplantation of FGSCs in chemotherapy-treated women, we designed and performed animal model experiments to mimic such cases in humans.

In our study, we successfully isolated the FGSCs from the mice with ovarian failure induced by CTx, transfected the FGSCs with GFP-carrying virus and transplanted them back to the females exposed to nonlethal doses of CTx. The CTx-exposed females with FGSC transplantation within one week successfully produced offspring, while CTx-exposed females without FGSCs transplantation were infertile. With the identification of morphology, gene expression and Southern blotting, we confirmed that FGSCs existed in ovarian failure mice and that they exhibited normal function to form oocytes in vivo. When fertilized, the oocytes could eventually produce progeny, which suggested that female cancer patients who suffered from ovarian damage may still experience restored ovarian function and fertility via their own FGSCs. In addition, we observed a few oocyte-like cells 30–45 μm in size in in vitro culture (data not shown). Additionally, the gene expression of Gdf9 was positive in our results, which suggested that FGSCs can spontaneously self-differentiate in vitro and was consistent with the results of Tilly et al.[9]. However, CTx-exposed females with FGSC transplantation two months after CTx showed infertility. The possible reason for this is that the microenvironment of ovaries was already destroyed two months after CTx, resulting in ovary atrophy [21]. Even after transplantation of FGSCs, it is difficult to reverse ovarian damage. Although FGSC transplantation two months later cannot save the fertility of these mice, we could still isolate a few FGSCs from the atrophic ovaries three months after CTx (data not shown). In addition, when the female mice received a CTx dose of LD50, even the injection of FGSCs within 1week could not restore fertility. The degree of ovarian damage and infertility are mainly dependent on the type of chemotherapy drugs and their dosage [22,23]. In general, the mechanism of cytotoxic treatment is to cause DNA damage, which will result in a balance between pro-apoptotic and anti-apoptotic effects in cells. When a high dose of CTx acted on the cells, they fail to repair DNA damage and thus undergo apoptosis [24]. Therefore, it is difficult to reverse the apoptosis caused by an LD50 of CTx, even with the injection of FGSCs. To explore whether FGSCs could restore the ovarian function of prepubertal female mice with CTx treatment, we transplanted the FGSCs into ovarian failure mice at 6-weeks old. The estrogen concentrations of Group 1A, 1B and 1C suggested that intraovarian transplantation of FGSCs at an adult age can also restore ovarian function. Taken together, the later the time of FGSC injection and the greater the CTx dose (regardless of the type of drug) the patients received, the worse the therapeutic effect of FGSCs.

Although the FGSCs we used in this study were isolated from mice with ovarian failure induced by CTx, we also isolated FGSCs from normal adult female mice for another study. Exogenous hFGSCs should be transplanted into women with ovarian failure to restore their ovarian function and fertility. Unfortunately, in contrast to autologous hFGSCs, exogenous hFGSCs are difficult to obtain, and immune rejection can easily take place. Therefore, for female cancer patients who suffer from CTx treatment and want to protect their ovarian function and fertility by FGSC transplantation, we recommend isolating the FGSCs from ovaries before CTx treatment, even though it is possible to isolate them after CTx. We also recommend the transplantation of FGSCs to be performed as early as possible after CTx treatment in order to achieve a better therapeutic effect.

In summary, we successfully isolated and purified FGSCs from mice with ovarian failure induced by CTx to restore ovarian function and eventually produce offspring for the first time, providing a new potential clinical treatment of ovarian failure and infertility in female cancer survivors. In addition, considering the two important factors (the timing and the dose) described above, the results of the therapeutic effect of FGSCs probably indicated that the sooner the transplantation of FGSCs after CTx and the lesser the CTx dose the patients received, the better the outcome of the treatment. Although we achieved the above results, the standardization and optimization of the transplantation procedure for FGSCs need to be established in the future.

## Supporting Information

S1 ARRIVE Checklist NC3Rs ARRIVE Guidelines Checklist 2014. (DOCX)
