# Supplementary material for: Intraovarian Transplantation of Female Germline Stem Cells Rescue Ovarian Function in Chemotherapy-Injured Ovaries
Source: PLoS One. 2015 Oct 2;10(10):e0139824. doi: 10.1371/journal.pone.0139824 (PMC4592213; doi:10.1371/journal.pone.0139824)

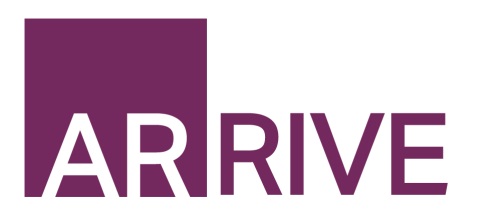


The ARRIVE Guidelines Checklist

Animal Research: Reporting In Vivo Experiments

Carol Kilkenny^1^, William J Browne^2^, Innes C Cuthill^3^, Michael Emerson^4^ and Douglas G Altman^5^

*^1^The National Centre for the Replacement, Refinement and Reduction of Animals in Research, London, UK, ^2^School of Veterinary Science, University of Bristol, Bristol, UK, ^3^School of Biological Sciences, University of Bristol, Bristol, UK, ^4^National Heart and Lung Institute, Imperial College London, UK, ^5^Centre for Statistics in Medicine, University of Oxford, Oxford, UK.*

|  | | ITEM | RECOMMENDATION | Section/ Paragraph |
| --- | --- | --- | --- | --- |
| 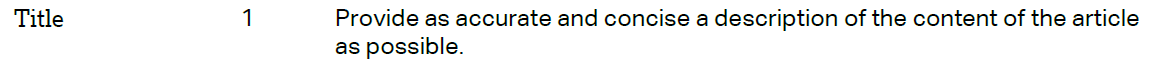 | | | Page 1 from line1-2 |  |
| 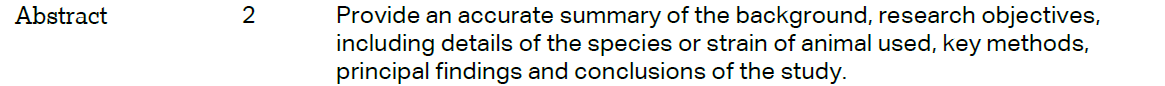 | | | Page 2 from line17-37 |  |
| INTRODUCTION | | |  |  |
| 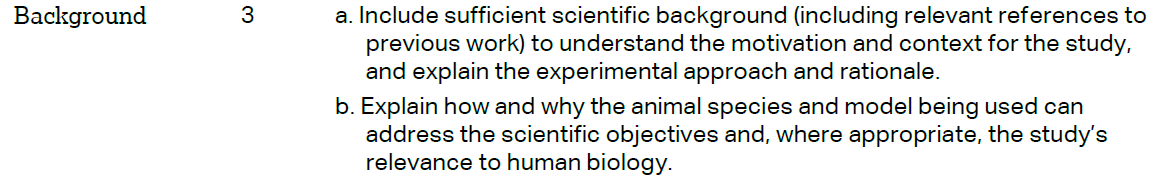 | | | Page3-4 from line 36-71 |  |
| 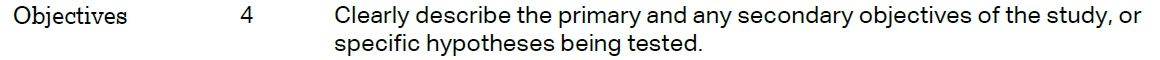 | | | Page 2 from line 28-31;page 4 from line 67-71 |  |
| METHODS | | |  |  |
| 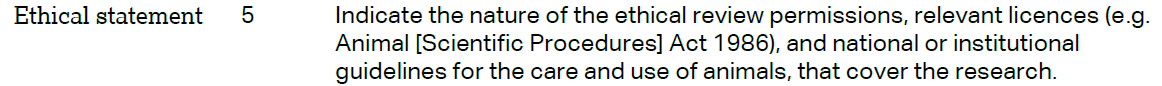 | | | Page 4 from 80-82 |  |
| 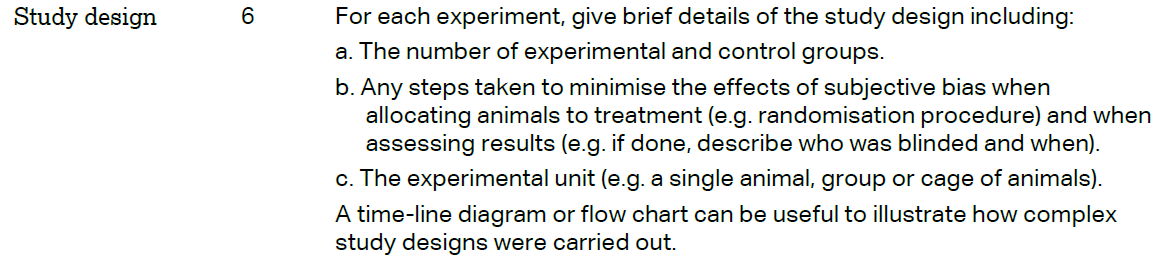 | | | Page 4 from line 74-80 |  |
| 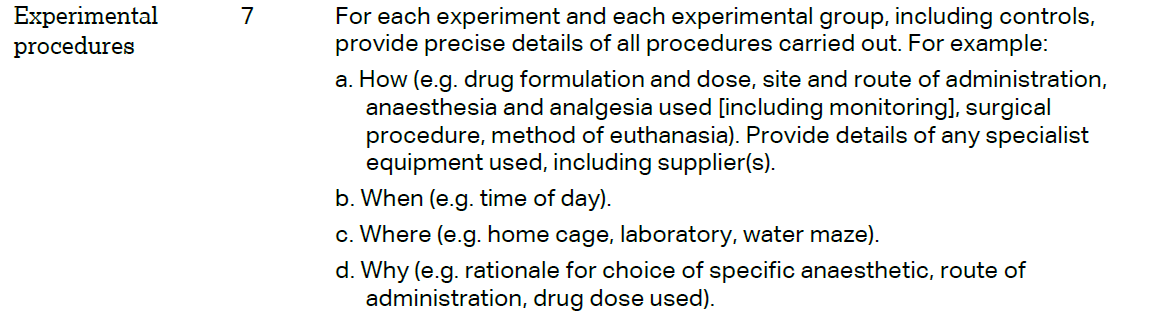 | | | Page 4 from line 74-80 |  |
| 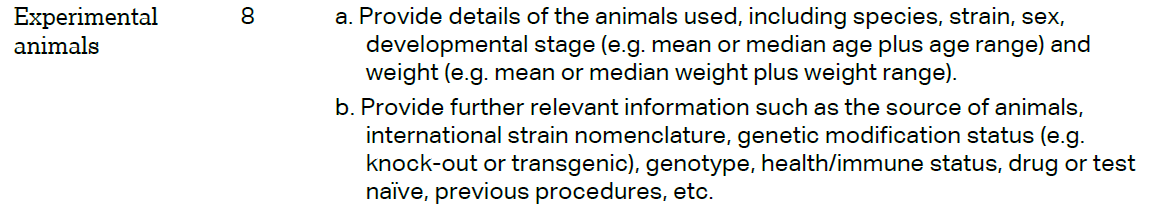 | | | Page 4 from line  74-80 |  |

The ARRIVE guidelines. Originally published in *PLoS Biology*, June 2010^1^

| 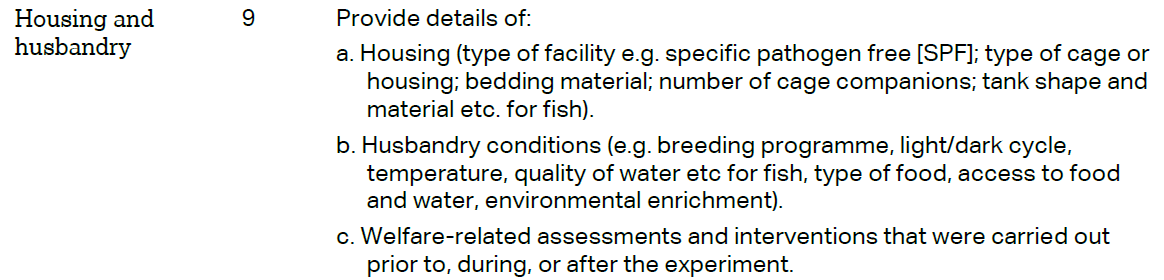 | Page 4 from line 76-77 | |
| --- | --- | --- |
| 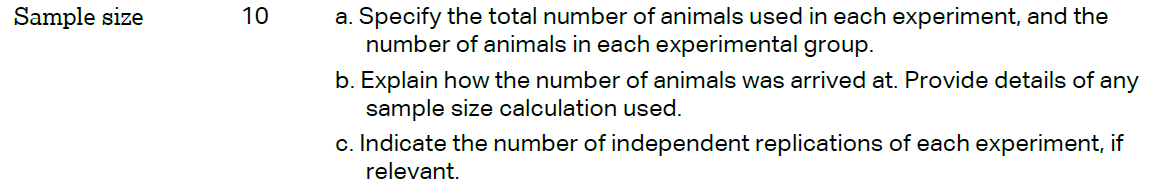 | Page11 from 223-253 | |
| 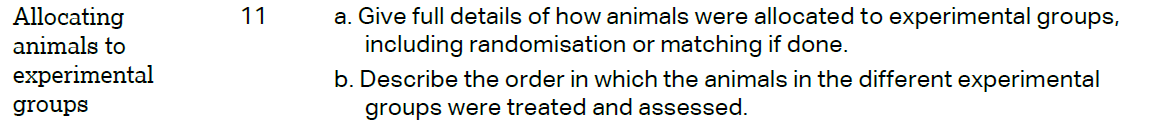 | Page11 from 223-253 | |
| 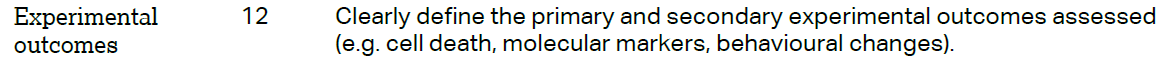 | Page11 from 223-253 | |
| 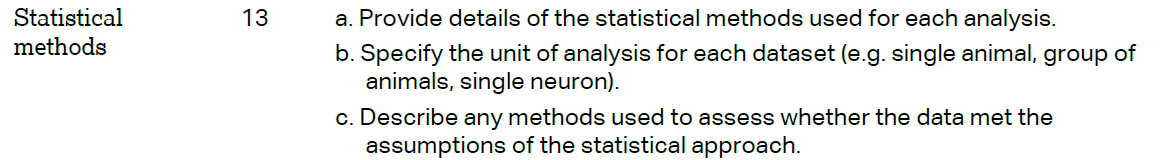 | Page 9 from 180-182 | |
| RESULTS |  | |
| 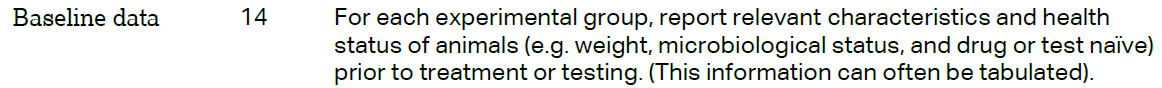 | Page11 from 223-253 | |
| 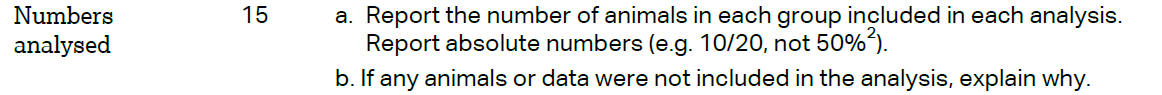 | Page11 from 223-253 | |
| 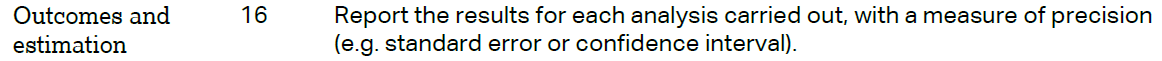 | Page11 from 223-253 | |
| 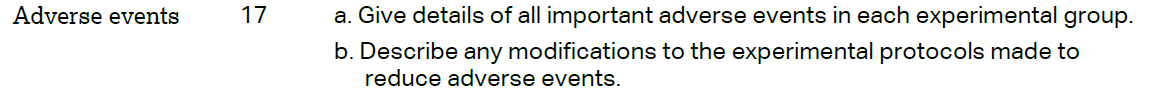 | N/A | |
| DISCUSSION |  | |
| 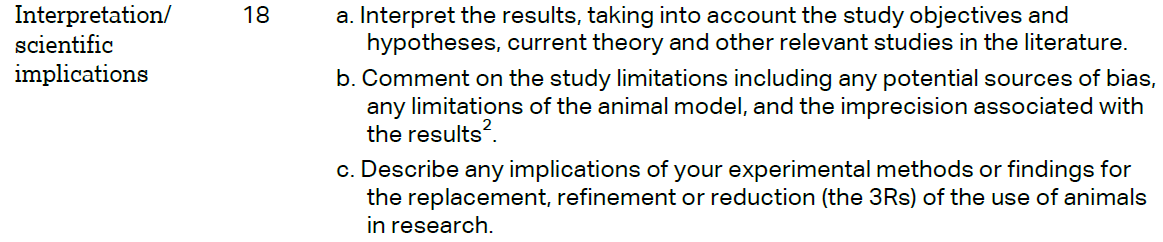 | Page 12-15 from line 255-303 | |
| 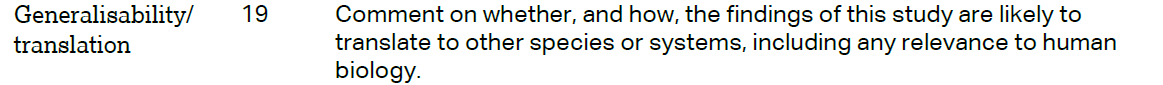 | Page 15 from line 304-314 | |
| 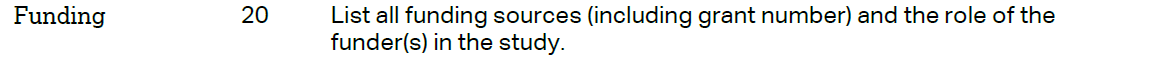 | | Showed at online submission |


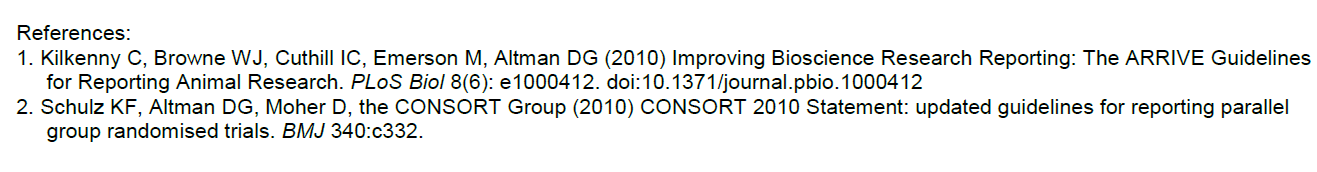

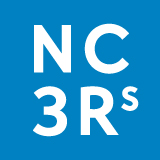

Supplement: S1 ARRIVE Checklist — (DOCX) [file pone.0139824.s001.docx]
